# Neuro-Behçet's Syndrome Without Genital Ulcers: A Case Report and Literature Review

**DOI:** 10.7759/cureus.64701

**Published:** 2024-07-16

**Authors:** Yongzhen Chen, Sumona Banerjee, Farid Khasiyev, Benjamin Kiaei, Sanhitha Valasareddy, Adam Kilian, Momina Soudagar Turkey

**Affiliations:** 1 Neurology, Saint Louis University School of Medicine, Saint Louis, USA; 2 Rheumatology, Saint Louis University School of Medicine, Saint Louis, USA

**Keywords:** genital ulcers, epididymitis, internuclear ophthalmoplegia, neuro-behçet's syndrome, behçet's disease

## Abstract

Behçet's disease is a rare multisystemic vasculitis characterized by oral ulcers, genital ulcers, and skin and ocular lesions. Neuro-Behçet’s syndrome is a condition in which individuals with Behçet's disease experience neurological symptoms that cannot be attributed to other neurological diseases. We present a rare case of neuro-Behçet’s syndrome with acute internuclear ophthalmoplegia and deteriorating neurological function with a prior history of recurrent oral ulcers with pathergy-like features, acneiform papulopustular rash, retinal hemorrhages, and recurrent epididymitis without genital ulcers. Patient improved with cyclophosphamide. This case underscores the importance of diagnosing and managing neuro-Behçet's syndrome in the absence of genital ulcers.

## Introduction

Behçet's disease is a rare, chronic relapsing multisystemic inflammatory variable vessel vasculitis that most commonly affects adults between the ages of 20 and 40 with the highest prevalence occurring in Turkey, Iran, and Japan and regions distributed along the “Silk Route” from the Mediterranean region to Asia [1]. Oral ulcers that recur frequently are observed in 97%-100% of patients during the diagnosis [2]. The presence of oral ulcers is crucial in diagnosing Behçet's disease, according to the International Study Group (ISG) diagnostic criteria [3]. Other frequent symptoms of the disease include genital ulcers (57%-96%), skin lesions (40-90%), and ocular lesions (37%) [4-6]. Although epididymitis is less common, it may still occur [7].

Neuro-Behçet’s syndrome is a condition in which individuals with Behçet's disease experience neurological symptoms that cannot be attributed to other neurological diseases [1]. This syndrome affects approximately 9% of patients with Behçet's disease, although it can range from 3% to 30% [8]. The most common type of neurological involvement in neuro-Behçet’s syndrome is central nervous system (CNS) parenchymal involvement, which may present with sensorimotor symptoms, cranial nerve palsy, cerebellar ataxia, headache, seizure, movement disorders, acute confusion state, psychiatric manifestations, and dementia [8]. Since neurological involvement in Behçet's disease is associated with a higher mortality rate [1], prompt assessment, and timely treatment are critical. This case report describes the diagnosis of neuro-Behçet's syndrome in a patient presenting with acute internuclear ophthalmoplegia and deteriorating neurological function with a prior history of recurrent oral ulcers with pathergy-like features (provoked by minor trauma such as tongue-biting), acneiform papulopustular rash on the trunk, limbs, and face, retinal hemorrhages, and recurrent epididymitis without genital ulcers.

This article was previously presented as a meeting abstract at the 2024 American Academy of Neurology (AAN) Annual Meeting on April 16, 2024.

## Case presentation

A 25-year-old male presented to the Emergency Department for a one-day history of double vision, mild right-sided weakness, nausea, and vomiting.

Prior history noted recurrent episodes of epididymitis associated with fever and night sweats, recurrent oral ulcers, thrush, unintentional weight loss, migraine headaches without aura, and iron-deficiency anemia. Patient noted that antibiotics only partially improved his episodes of epididymitis and that he had not been sexually active in the past eight years. He noted multiple oral ulcers in the past on his tongue, hard palate, uvula, throat, and lower lip. The ulcers lasted for several weeks before they resolved, and he only had a few days per month between the episodes when he was ulcer-free. He also reported unintentional weight loss of 20 pounds in nine months. Behçet's disease was considered in the past but was ruled out due to the absence of genital ulcers.

Physical exam revealed an inability to adduct the right eye, left eye abducting nystagmus, tongue deviation to the left, hyperreflexia of all extremities, and right lower extremity weakness. Patient had one tender right-sided oral ulcer on his tongue. Skin findings included acneiform pustules on the face and trunk. The ophthalmologic exam revealed multiple peripheral dot blot hemorrhages in the left eye.

**Figure 1:**
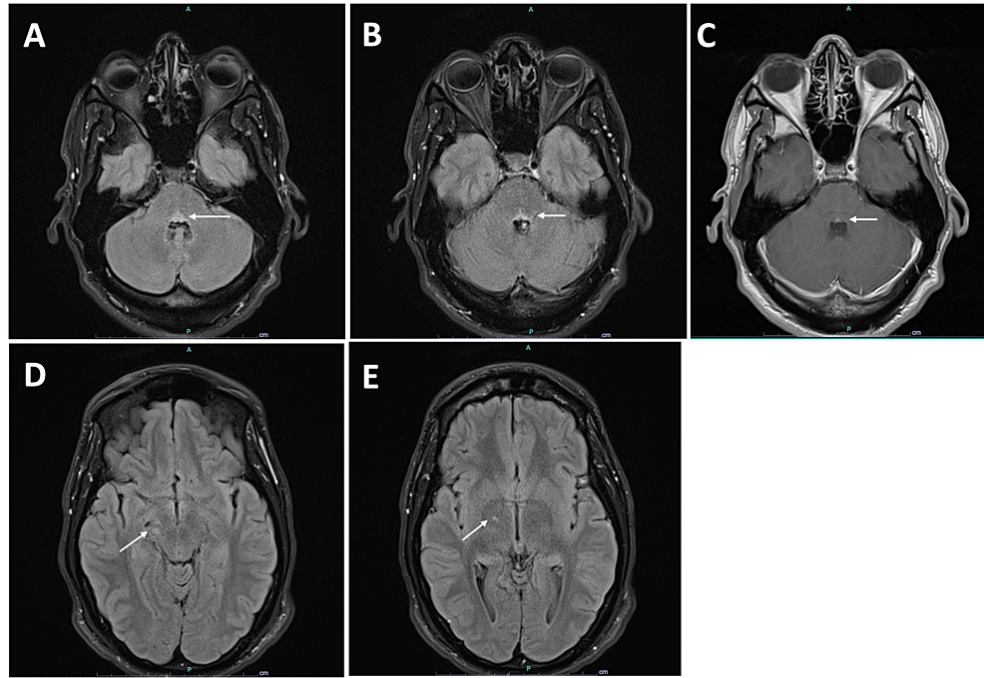
Brain MRI at initial presentation A, B: hyperintensity in the pontine tegmentum along the dorsal pons in the fluid attenuated inversion recovery (FLAIR) sequence. C: subtle contrast enhancement in the dorsal pons in the post-contrast T1 sequence. D: mild hyperintensity in the right cerebral peduncle in the FLAIR sequence. E: a small focus of hyperintensity in the right thalamo-capsular junction in the FLAIR sequence.

Laboratory testing of blood revealed a slightly elevated white blood cell count (11.7x10^3^/uL; normal 3.5-10.5x10^3^/uL) and absolute neutrophil (9.32x10^3^/uL; normal 1.60-7.00x10^3^/uL). The cerebrospinal fluid (CSF) analysis revealed glucose of 48 mg/dL (normal 40-70 mg/dL), protein of 37 (normal 15-45 mg/dL), white blood cells of 13x10^6^/L (normal 0-5x10^6^/L), with no bacteria/virus/fungus growth, negative herpes simplex virus 1 and 2, cryptococcus, venereal disease research laboratory (VDRL), West Nile, enterovirus, and normal protein electrophoresis panel. The oligoclonal band numbers matched those of CSF and serum (Tables 1, 2). Prior to hospitalization, the patient was tested for gonorrhea, chlamydia, HIV, Treponema pallidum antibody, and hepatitis B and C, and all results were negative. Brain MRI showed hyperintensity in the pontine tegmentum along the dorsal aspect of the pons, possibly involving the bilateral sixth nerve nuclei and the bilateral facial colliculi in the fluid attenuated inversion recovery (FLAIR) sequence with subtle contrast enhancement in the T1 sequence (Figures 1A-1C). There was mild hyperintensity in the right cerebral peduncle and a small focus of hyperintensity in the right thalamo-capsular junction in the FLAIR sequence (Figures 1D, 1E). Based on the patient's history of recurrent painful oral ulcers with pathergy, acneiform papulopustular rash, recurrent epididymitis, retinal hemorrhage suggestive of retinal vasculitis, hyperreflexia, pontine encephalitis on MRI, pleocytosis in CSF, a diagnosis of neuro-Behçet's syndrome was made. The patient was started on three days of pulse-dose IV methylprednisolone (1 g daily) therapy. A CT chest angiogram was negative for pulmonary artery aneurysm.

**Table 1:** Serum labs and cerebrospinal fluid (CSF) labs WBC: White blood cells RBC: Red blood cells VDRL: Venereal disease research laboratory test PCR: Polymerase chain reaction

	Values	Reference range and units
Serum labs		
WBC	11.7 x 10^3^	3.5 - 10.5 x 10^3^/uL
RBC	4.79 x 10^6^	4.30 - 5.70 x10^6^/uL
Hemoglobin	13.1	12.0 - 17.6 g/dL
Hematocrit	39.8	35.2 - 51.7 %
Platelet Count	339 x 10^3^	150 - 400 x 10^3^/uL
Neutrophils Absolute	9.32 x 10^3^	1.60 - 7.00 x 10^3^/uL
C-Reactive Protein	5.2	<=0.5 mg/dL
Erythrocyte Sedimentation Rate	56	0 - 15 MM/HR
CSF Labs		
Glucose	48	40 - 70 mg/dL
Protein	37	15 - 45 mg/dL
WBC	13x10^6^	0 - 5 x10^6^/L
Lymphocytes % Fluid	82	%
Monocytes % Fluid	10	%
Herpes Simplex Virus 1 and Virus 2 PCR	Not detected	Not detected
Cryptococcus Antigen	Negative	Negative
West Nile IgG Ab	0.39	<=1.29 IV
West Nile IgM Ab	0.00	<=0.89 IV
Enterovirus by PCR	Not detected	Not detected, Indeterminate
QuantiFERON TB Gold Plus	Negative	Negative
VDRL	Non Reactive	Non Rea:<1:1
Oligoclonal Bands Number	Matching	N/A
Viral, fungus, gram stain culture	Negative	Negative

**Table 2:** Rheumatologic labs ANA: Antinuclear Antibodies ANCA: Antineutrophil Cytoplasmic Antibodies NMO/AQP4 FACS: Neuromyelitis Optica/Aquaporin-4-IgG Fluorescence-Activated Cell Sorting Titer Assay MOG FACS: Myelin Oligodendrocyte Glycoprotein Fluorescence-Activated Cell Sorting Assay

	Values	Reference range and units
ANA IgG	Not detected	Not detected
ANCA pattern IFA	Not detected	Not detected
NMO/AQP4 FACS, Serum	Negative	Negative
MOG FACS, Serum	Negative	Negative
Ganglioside (GM1, GD1b and GQ1b) Antibodies, IgG and IgM	Negative	Negative
SSA-52 (Ro52) and SSA-60 (Ro60) antibodies	Negative	Negative
Vitamin B12	808	213 - 816 pg/mL
HLA-B51	Not detected	Not detected

**Figure 2:**
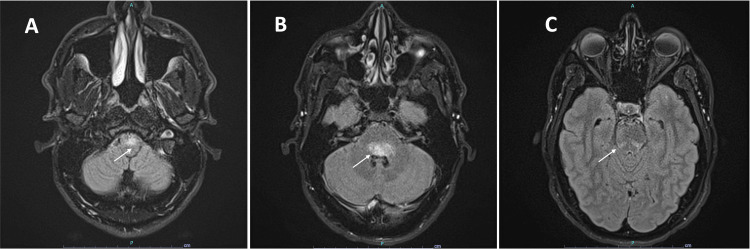
Repeat brain MRI on Day 4 of methylprednisolone treatment Repeat brain MRI on Day 4 of methylprednisolone treatment showing hyperintensities in the anterior medulla, dorsal pons, and dorsal midbrain in the fluid attenuated inversion recovery (FLAIR) sequence compared to MRI at initial presentation.

During the patient's hospital course, his symptoms progressed with worsening right-sided weakness, new distal sensory loss, unilateral ataxia, and a new dot blot hemorrhage with cotton wool spots in the macula of the left eye. A repeat MRI of the brain revealed the progression of hyperintensities in the medulla, dorsal pons, and dorsal midbrain/tegmentum, extending into the right cerebral peduncle and right basal ganglia in the FLAIR sequence (Figure 2A-2C). Thus, the course of pulse-dose methylprednisolone treatment was extended for one more day (a total of four days). Due to worsening neurologic findings, the patient was administered intravenous cyclophosphamide 600 mg/m^2^ (1400 mg) with plans to continue cyclophosphamide IV monthly for three to six months outpatient. He was transitioned to prednisone 60 mg with a plan to start adalimumab and discharged once symptoms improved.

## Discussion

Behçet's disease is a rare chronic relapsing multisystemic inflammatory variable vessel vasculitis that typically presents with mucocutaneous symptoms such as orogenital ulcers and skin lesions [9]. The involvement of other organs, such as the musculoskeletal system, eyes, nervous system, gastrointestinal tract, and cardiopulmonary system can lead to significant morbidity and mortality [9]. The exact pathogenesis of Behçet's disease is still being clarified, but genetic and environmental factors are believed to play a role [9].

The diagnosis of Behçet's disease can be made through the ISG criteria or the International Criteria for Behçet's Disease (ICBD) [3,10]. According to the ISG criteria, a patient must have oral aphthosis and two out of four other symptoms (genital aphthosis, skin manifestations, ocular manifestations, or pathergy) to be diagnosed with Behçet's disease [3]. The ICBD requires three or more symptoms, such as genital aphthosis, ocular lesions, oral aphthosis, skin lesions, vascular lesions, neurological lesions, and pathergy, to be present for the diagnosis [10].

In our patient's case, he had recurrent oral ulcers, acneiform lesions, and ocular manifestations, so he met the ISG diagnostic criteria. Research has suggested that pathergy test can be replaced with other features such as epididymitis, aseptic meningoencephalitis, cerebral vasculitis, recurrent phlebitis, arteritis, synovitis, or focal bowel ulceration, especially in Northern European and North American populations where pathergy is less common [11]. Our patient also satisfied the ICBD classification with recurrent oral ulcers, ocular lesions, skin lesions, and CNS involvement.

Since oral ulcers are the most common manifestation of Behçet's disease, it is vital to exclude other causes of oral ulcers such as infection, cyclical neutropenia, medication side effects, inflammatory bowel disease (IBD), vitamin B12 deficiency, rheumatic disease or periodic fever, aphthosis, pharyngitis, and adenitis (PFAPA) [9]. It is also essential to rule out other possible causes of genital ulcerations, such as sexual infections, IBD, drug reactions, trauma, and neoplasms [9]. The combination of both oral and genital ulcers can be due to mouth and genital ulcers with inflamed cartilage (MAGIC) syndrome, cyclical neutropenia, and adverse drug effects, which must also be considered [9].

Neuro-Behçet's syndrome is a rare condition with neurological symptoms in patients who meet the diagnostic criteria for Behçet's disease [12]. The syndrome is more common in male patients and can affect 5-50% of patients with Behçet's disease, depending on the geographic region [12]. The CNS is the most commonly affected area in neuro-Behçet's syndrome, and it can be divided into two categories: parenchymal and non-parenchymal [12]. In imaging tests, lesions of neuro-Behçet's syndrome are typically found in the brainstem, and they may extend to the diencephalon and basal ganglia [12]. These lesions are different from the periventricular lesions seen in multiple sclerosis [12]. However, chronic parenchymal lesions can be similar to those found in multiple sclerosis and may be challenging to differentiate [13]. About 70-80% of neuro-Behçet's syndrome cases with parenchymal involvement show CSF findings, which include elevated CSF protein and normal glucose levels [13]. CSF cell count is elevated in 60-80% of cases, and it may show associated neutrophilia, lymphocytosis, or mixed cellularity [13]. Unlike multiple sclerosis, it is rare for neuro-Behçet's syndrome to have intrathecal IgG synthesis, including oligoclonal IgG bands and elevated CSF IgG levels [12]. Neurophysiology testing may be helpful if peripheral nervous system or optic nerve involvement is suspected [13]. Other conditions such as infections, systemic lupus erythematosus, Sjogren's syndrome, and malignancies can cause similar symptoms and should be ruled out before diagnosing neuro-Behçet's syndrome [12].

The main objective of treating Behçet's disease and neuro-Behçet's syndrome is to reduce inflammation quickly and prevent irreparable organ damage. Corticosteroids are often the initial treatment for Behçet's disease, and other medications such as colchicine, azathioprine, tumor necrosis factor-alpha inhibitor, cyclophosphamide, and others may also be used for induction or maintenance therapy [14]. Although there is no controlled clinical trial data on treating neuro-Behçet’s syndrome due to the rarity of the disease, most clinicians treat acute presentations with IV methylprednisolone, followed by a slow taper of oral steroids [13]. Retrospective studies have shown that two-thirds of patients respond to steroids, while the other third experience relapses of disease or a progressive course [13]. Our patient had worsening neurologic findings after three days of pulse dose steroid therapy. Therefore, cyclophosphamide was started for CNS penetration with plans to repeat. Poor prognostic factors include brainstem or spinal cord presentation, frequent relapses, early disease progression, and high CSF pleocytosis [15].

Our neuro-Behçet's case is unique in two aspects: first, the patient presented without genital ulcers, the second most common symptom of Behçet's disease [5]. The absence of genital ulcers may distract practitioners from diagnosing Behçet's disease, although it is not a requirement for diagnosing Behçet's disease. Very few case reports of Behçet's disease or neuro-Behçet's syndrome had no concurrent or past history of genital ulcers at diagnosis [4]. Genital ulcers were not seen in these cases, likely because they were usually not the first manifestation of the disease [6]. Second, our patient has a history of recurrent epididymitis, a rare manifestation of Behçet's disease [7]. A study of 780 male patients with Behçet's disease showed a prevalence of epididymitis of 4.6% [7]. Two of the 36 patients with a history of epididymitis or concurrent epididymitis had CNS involvement (5.6%) [7]. Patients with Behçet's disease and epididymitis are more likely to have CNS involvement compared to those without epididymitis [7]. Other studies have shown that the prevalence of epididymo-orchitis in Behçet's disease varies across different regions and age groups. Middle Eastern countries such as Jordan and Iraq have a higher prevalence (27-31%) than Mediterranean and European countries (10-20%) and East Asian countries (0.6-6%) [16]. Juvenile patients (25%) have a higher frequency than adults (8.9%) [16].

## Conclusions

Although genital ulceration is the second most common symptom of Behçet's disease, its presence is not required for diagnosis. In cases where patients exhibit CNS involvement with normal serum and CSF labs, recurrent oral ulcers, cutaneous lesions, ocular lesions, and epididymitis, even in the absence of genital ulcers, neuro-Behçet's syndrome should be considered. This is particularly important when alternative neurological disorders and infections have been ruled out. Diagnosing and managing neuro-Behçet's syndrome through multidisciplinary collaboration between rheumatology and neurology is recommended.
